# Welcome from the policies, socio-economic aspects, and health systems research section

**DOI:** 10.1186/s40608-015-0052-6

**Published:** 2015-05-28

**Authors:** Anna Peeters, Helen L Walls, Kathryn Backholer, Gary Sacks, Asnawi Abdullah

**Affiliations:** Obesity & Population Health, Baker IDI Heart and Diabetes Institute, Melbourne, Australia; Department of Epidemiology and Preventive Medicine, Monash University, Melbourne, Australia; London School of Hygiene and Tropical Medicine, London, UK; Leverhulme Centre for Integrative Research on Agriculture and Health, London, UK; WHO Collaborating Centre for Obesity Prevention, Deakin University, Melbourne, Australia; Faculty Public Health, University Muhammadiyah, Aceh, Indonesia

## Abstract

At BMC Obesity, the Policies, Socio-economic Aspects, and Health Systems Research Section provides an opportunity to submit research focussed on what we need to know to support implementation of obesity policies most likely to achieve substantial, sustainable and equitable reductions in the prevalence of obesity globally. Here, we present the aims and objectives of this section, hearing from each of the Associate Editors in turn. The ambition of the Policies, Socio-economic Aspects, and Health Systems Research Section is to foster innovative research combining scientific quality with real world experience. We envisage this will include research addressing the structural drivers of obesity, solution oriented research, research addressing socio-economic inequalities in obesity and obesity prevention in low and middle income countries. We look forward to stimulating research to advance both the methods and substance required to drive uptake of effective and equitable obesity reduction policies globally.

## Introduction

We are entering into an exciting period for prevention of obesity. There is increasing global acceptance of the likely negative impact of obesity trends on population health, wellbeing and productivity. We are starting to see consideration and implementation by governments of a range of obesity prevention policies addressing healthy eating and physical activity in a number of contexts. Additionally, many communities, although most often in high-income countries (HICs), are expressing high levels of support for government and industry action to improve our access to healthy food and activity options and to decrease the availability of unhealthy options. The recent formation of the World Health Organisation’s Commission on Ending Childhood Obesity is intended to drive further action. But we still have a long way to go before we see widespread implementation of the comprehensive and complementary suite of interventions that will likely be needed to make a substantive and lasting impact on our high and increasing obesity rates. At BMC Obesity, in the Policies, Socio-economic Aspects, and Health Systems Research Section we are looking for research that will advance our ability to support implementation of obesity prevention policies most likely to achieve substantial, sustainable and equitable reductions in the prevalence of obesity globally. Here a number of the Section’s Associate Editors describe their vision of what will be needed. While this is not an exhaustive list it provides a glimpse of some of the steps forward that we hope to showcase in the coming issues of BMC Obesity.

### Addressing the structural drivers of obesity (Helen Walls)

Addressing the ‘upstream’ or structural determinants that shape obesity risk, sometimes called the ‘causes of the causes’, is a likely target of much needed ‘solutions-oriented research’. Actions addressing obesity risk at the individual level will remain important, as later discussed, particularly for those at high risk of weight gain or with established disease, but some of the important structural determinants of obesity can only be addressed at a collective or population level. Furthermore, interventions at the population level that shape healthier lives often achieve greatest benefits at lowest cost, and are easier to sustain over time [1].

Some key examples of these structural determinants include urban design, poverty and inequality, unemployment, agriculture and food systems, and trade liberalisation, as all have significant influence over our lifestyle choices and behaviors. These areas are receiving increasing attention from researchers, but there is considerable scope for further evidence. In BMC Obesity we welcome research examining the influence of these determinants on obesity risk, and how to improve governance in these areas, including institutional structures and appropriate multi—and cross-sectoral policy—making in different country settings.

Agriculture and food systems, for example, are key determinants of nutrition and related health, including obesity, but do not always contribute to positive nutritional outcomes. There is a recognised urgent need for agriculture and food systems to be more ‘nutrition sensitive’ [2, 3], and much scope for research to help elucidate the impact of agriculture and food systems on nutrition, obesity and non-communicable disease, and to address how best to achieve appropriate changes in agriculture and food systems.

Trade liberalisation is another recognised driver of nutrition, obesity and non-communicable disease [4–6]. The multilateral trade agreements negotiated through the General Agreement on Tariffs and Trade, the World Trade Organization and increasingly, bilateral and regional trade agreements, have facilitated increased international trade and foreign direct investment (FDI) in foods, declining protection of domestic industry, and the globalization of food advertising [5, 6]. These changes have resulted in the increased influence of multinational and transnational corporations, and increased availability of ultra-processed food and sugar-sweetened beverages [7]. Furthermore, modern trade agreements are increasingly including clauses allowing greater industry and intellectual property protections, with potentially far-reaching implications for public health [8, 9].

Noting this, we are interested in research that examines how trade liberalisation affects the food supply chain, evaluates the impact of trade policy on nutrition and obesity risk, and helps us to understand better the practices of the food and beverage industry and how it shapes our behavior. We also welcome research examining how, in the context of increasing trade liberalization, public health actions to address consumption of ultra-processed food and sugar-sweetened beverages can be strengthened. Another key area of interest is research examining how these issues differ between low-, middle—and high-income countries, including the differential public health effects of trade agreements. Such research is likely to come from diverse fields, including economics, trade and health law, nutrition, marketing and psychology.

Effective obesity interventions will likely involve changes to policy and regulatory environments at global, regional, national and local levels, and will include sectors outside of what is traditionally considered health. However, it can be difficult for government agencies outside health to respond to health-oriented policies [10], and developing policy coherence between sectors is often hampered by differences in sectoral ‘frames’ or worldviews [7]. Research that examines how to generate shared understandings of problems, opportunities and solutions in regard to addressing obesity will be welcomed. It can also be difficult for politicians to support structural changes that might be perceived to threaten certain powerful industries, for example the food industry. We are interested in research that examines the governance structures and mechanisms that may constrain the ability of countries and health authorities to respond to health problems, and research that examines the characteristics and supporting features of those cross- and multi-sectoral interventions that *have* been achieved.

### Solution-oriented research (Gary Sacks)

It is well recognised that broad-ranging actions will be needed to address obesity [11]. This will need to include government policy action across multiple sectors and levels of government, including a range of policy instruments such as regulations, legislation, programs and advocacy [12]. It may also include changes to governance and institutional structures in order to facilitate coordinated action [13]. While many question the appropriate role for the private sector in obesity prevention [14], it is also widely acknowledged that the food industry, sporting goods manufacturers and suppliers, advertisers, public relations firms, private health insurers and the media, can also play important roles in obesity prevention at the population-level [11].

Much of the existing obesity research is focused on identifying underlying causes and correlates of obesity-related risk factors and diseases. While this research continues to be important in understanding the problem, we welcome submissions that are focused on what can be done to solve the problem, particularly actions that can be applied at the population level. This ‘solution-oriented’ research [15] is likely to have immediate relevance to policy and can directly inform public health action. This includes research into all aspects of the policy process, including the way in which policy is developed and implemented, the impact of policies on food and physical activity environments and health outcomes, and economic evaluation of policy actions. Examination of novel policy approaches, including regulatory and non-regulatory options, and including both government and private sector policies, will be particularly welcome. Submissions can relate to empirical and modelled studies, in recognition that it is not always necessary to wait for evaluations of real-world implementations in order to produce evidence that can inform decision makers.

Given the low-levels of implementation of obesity prevention actions globally [16], we are also interested in research into mechanisms for improving the accountability of governments and the private sector in this area. Similarly, research into novel approaches to encouraging the private sector to invest in obesity prevention actions and increase their transparency will be welcome. We will value research that has a strong focus on knowledge translation and exchange with decision-makers.

### Socio-economic inequalities in obesity (Kathryn Backholer)

The prevalence of obesity follows a socio-economic gradient. For high income countries this gradient is generally negative; a higher education, income and/or living in a more affluent neighbourhood is associated with a lower risk of adverse weight gain [17–19]. These gradients are commonly more striking for women and children [20, 21]. For low and middle income countries (LMICs) the socio-economic gradient is more variable. For many of these countries, the burden of obesity is greater for those with more affluence, however as globalization oversees the change from traditional diets and lifestyles to more westernised models (a phenomenon known as the nutritional transition), we have witnessed shifts to a greater burden of obesity from higher to lower socio-economic groups [22]. At BMC Obesity, we are particularly keen to understand the burden of obesity and highlight possible solutions for groups that are disproportionately represented in the obesity epidemic, such as those with a lower socio-economic position.

In view of the positive risk relationships between obesity and a large number of co-morbid conditions [23, 24], these socio-economic inequalities in obesity are likely to contribute to inequalities in the population burden of non-communicable diseases. Failing to prioritise the equitable prevention, management and treatment of obesity risks widening disparities in non-communicable disease. To ensure health equity remains a central focus in all decision making it is essential that socio-economic inequalities in the population prevalence of obesity is systematically and routinely monitored. We therefore welcome analyses of recent trends in population weight disaggregated by socio-economic position and descriptive analyses of the health burden of such unequal trends. This will be particularly important for LMICs that are at the height of economic and nutritional transitions.

It is further essential that strategies to reduce socio-economic inequalities in obesity are identified, prioritised and implemented. A vast range of obesity interventions and policies have been suggested, but too often the effect of such are not evaluated across socio-economic strata [25, 26]. We therefore welcome solutions-oriented research that applies empirical, theoretical and/or policy analyses to illuminate the evidence on what works and what does not work, both at reducing weight across the socio-economic gradient and for interventions specifically targeted to socio-economic groups, where the burden of obesity is greater. This will importantly include strategies that address the structural social determinants of health, or the ‘causes of causes’, as noted in previous sections of this editorial. Nevertheless, it is recognised that a health equity evaluation of all types of interventions, from those aimed at the individual to those aimed at the population level, will be a valuable contribution to the evidence base. We additionally seek research that presents the opportunities and challenges for the equitable prevention, management and treatment of obesity. What are the barriers and facilitators to effective and equitable interventions? And how can we capitalise on this knowledge to ensure everyone benefits from obesity prevention, regardless of their socio-economic position within society?

Identifying effective and equitable obesity interventions is also somewhat limited by our understanding of the specific environmental, behavioural and psychosocial drivers of such inequalities. We encourage articles that elucidate these specific drivers, at both individual and societal levels, and the relative importance of such, so that leverage points for intervention can be identified and prioritised.

### Obesity prevention in low—and middle-income countries (Asnawi Abdullah)

The obesity epidemic was first noted in high-income countries in the 1960s and 1970s, but in recent decades most low—and middle-income countries have also experienced a rapidly increasing prevalence in obesity [27, 28]. Thus, in many of these countries overweight and obesity co-exist alongside a significant prevalence of underweight, a situation which has been termed the ‘double burden’ of malnutrition [29]. This double burden is reported to occur not just in the same population/community, but in the same household/family, or even in the same person/individual [30, 31]. In South Asia, for example, whilst overweight and associated non-communicable diseases are on the rise, underweight remains common, affecting for example a third of Bangladeshi women [32, 33].

Research examining the underlying drivers of changing body weight trends and obesity in LMICs is a priority, and we welcome submissions in this area. The potential drivers are many and varied, and given the double burden of malnutrition described above, we would welcome work examining the cause and factors associated between co-existing underweight and overweight and obesity. Another key area in need of greater examination is the role of corporations in driving changes in dietary patterns and associated obesity in LMICs. Corporations often move quickly and exploit regulatory gaps, many of which are to be found in countries with weaker institutions such as in many LMICs, and quickly emerge for example in formerly closed countries such as the emerging USSR in the 1990s and more recently, Myanmar [1].

Importantly, strategies to address the burgeoning prevalence of overweight and obesity in LMICs need to be context-specific. A ‘one-size-fits-all’ approach from HICs is unlikely to provide the answer. Policies designed in high-income settings should not be assumed to work in lower-income settings, and any transfer of policies must take account of cultural norms [1]. We are interested in research discussing the particular challenges and opportunities to addressing and managing obesity in LMICs.

There are also many other aspects of obesity in LMICs in which we welcome submissions. For example, as described in a previous section of this editorial, within HICs, higher obesity prevalence is observed in rural areas and among people with a lower socioeconomic position, however this is often reversed in LMICs. Emerging evidence suggests that with nutrition and epidemiological transitions underway, these patterns are changing [27]. In HICs there is a considerable body of evidence documenting trends and patterns in population body weight, and such work has been vital for understanding the dynamics of the obesity epidemic, and for informing the design of interventions. In many LMICs the dynamics of changes in body weight, including by population sub-groups, are not well documented. We welcome submissions addressing this important area. Furthermore, there remains controversy over the appropriate definition and cut-off points for particular body weight categories in different populations [34], another area in which we welcome submissions.

In sum, we welcome research on obesity of particular relevance to LMICs, including submissions on the aetiology of obesity in these settings, the analysis of trends and patterns, and the various issues relevant to selecting and implementing appropriate prevention strategies.

## References

[1] McKee M, Haines A, Ebrahim S, Lamptey P, Barreto ML, Matheson D (2014). Towards a comprehensive global approach to prevention and control of NCDs. Globalization Health.

[2] Ruel MT, Alderman H (2013). Maternal, Child Nutrition Study G. Nutrition-sensitive interventions and programmes: how can they help to accelerate progress in improving maternal and child nutrition?. Lancet.

[3] Hawkes C, Thow AM, Downs S, Ghosh-Jerath S, Snowdon W, Morgan EH, et al. Leveraging agriculture andfood systems for healthier diets and non-communicable disease prevention: The need for policy coherence. Expert working paper prepared for FAO’s Global Forum on Food Security and Nutrition online discussion, “Nutrition-enhancing agriculture and food systems,” 2013 [28/04/2015]. Available from: http://www.fao.org/fileadmin/user_upload/agn/pdf/2-pager_Hawkes.pdf

[4] Blouin C, Chopra M, van der Hoeven R (2009). Trade and social determinants of health. Lancet.

[5] Rayner G, Hawkes C, Lang T, Bello W (2006). Trade liberalization and the diet transition: a public health response. Health Promot Int.

[6] Thow AM (2009). Trade liberalisation and the nutrition transition: mapping the pathways for public health nutritionists. Public Health Nutr.

[7] Baker P, Kay A, Walls H (2014). Trade and investment liberalization and Asia’s noncommunicable disease epidemic: a synthesis of data and existing literature. Globalization Health.

[8] Friel S, Gleeson D, Thow AM, Labonte R, Stuckler D, Kay A (2013). A new generation of trade policy: potential risks to diet-related health from the trans pacific partnership agreement. Globalization Health.

[9] Gleeson D, Friel S (2013). Emerging threats to public health from regional trade agreements. Lancet.

[10] Walls HL, Pearce N. Globalisation and the impact on non-communicable diseases. In: Hanefeld J, editor. Globalization and health: Berkshire, England. Open University Press. 2nd edition; 2014.

[11] World Health Organization (2013). Global Action Plan for the Prevention and Control of Non-communicable Diseases 2013–2020.

[12] Sacks G, Swinburn B, Lawrence M (2009). Obesity Policy Action framework and analysis grids for a comprehensive policy approach to reducing obesity. Obes Rev.

[13] Magnusson RS (2008). What’s law got to do with it Part 2: Legal strategies for healthier nutrition and obesity prevention. Australia New Zealand Health Policy.

[14] Moodie R, Stuckler D, Monteiro C, Sheron N, Neal B, Thamarangsi T (2013). Profits and pandemics: prevention of harmful effects of tobacco, alcohol, and ultra-processed food and drink industries. Lancet.

[15] Robinson TN, Sirard JR (2005). Preventing childhood obesity: a solution-oriented research paradigm. Am J Prev Med.

[16] Swinburn B, Kraak V, Rutter H, Vandevijvere S, Lobstein T, Sacks G, et al. Strengthening accountability systems to create healthy food environments and reduce global obesity. Lancet. 2015;(in press). 10.1016/S0140-6736(14)61747-5 25703108

[17] McLaren L (2007). Socioeconomic status and obesity. Epidemiol Rev.

[18] Ball K, Crawford D (2005). Socioeconomic status and weight change in adults: a review. Soc Sci Med.

[19] Devaux M, Sassi F (2013). Social inequalities in obesity and overweight in 11 OECD countries. Eur J Public Health.

[20] Ogden CL, Lamb MM, Carroll MD, Flegal KM (2010). Obesity and socioeconomic status in children and adolescents: United States, 2005–2008. NCHS data brief.

[21] Wardle J, Waller J, Jarvis MJ (2002). Sex differences in the association of socioeconomic status with obesity. Am J Public Health.

[22] Dinsa GD, Goryakin Y, Fumagalli E, Suhrcke M (2012). Obesity and socioeconomic status in developing countries: a systematic review. Obesity.

[23] Guh DP, Zhang W, Bansback N, Amarsi Z, Birmingham CL, Anis AH (2009). The incidence of co-morbidities related to obesity and overweight: a systematic review and meta-analysis. BMC Public Health.

[24] Haslam DW, James WP (2005). Obesity Lancet.

[25] Beauchamp A, Backholer K, Magliano D, Peeters A (2014). The effect of obesity prevention interventions according to socioeconomic position: a systematic review. Obesity reviews.

[26] Magnee T, Burdorf A, Brug J, Kremers SP, Oenema A, van Assema P (2013). Equity-specific effects of 26 Dutch obesity-related lifestyle interventions. Am J Prev Med.

[27] Popkin BM, Adair LS, Ng SW (2012). Global nutrition transition and the pandemic of obesity in developing countries. Nutr Rev.

[28] Swinburn BA, Sacks G, Hall KD, McPherson K, Finegood DT, Moodie ML (2011). The global obesity pandemic: shaped by global drivers and local environments. Lancet.

[29] Kolcic I (2012). Double burden of malnutrition: A silent driver of double burden of disease in low- and middle-income countries. J Global Health.

[30] Doak CM, Adair LS, Bentley M, Monteiro C, Popkin BM (2005). The dual burden household and the nutrition transition paradox. Int J Obes (Lond).

[31] Garrett JL, Ruel MT (2005). Stunted child-overweight mother pairs: prevalence and association with economic development and urbanization. Food Nutr Bull.

[32] Ahmed T, Mahfuz M, Ireen S, Ahmed AM, Rahman S, Islam MM (2012). Nutrition of children and women in Bangladesh: trends and directions for the future. J Health Popul Nutr.

[33] Ghaffar A, Reddy KS, Singhi M (2004). Burden of non-communicable diseases in South Asia. BMJ.

[34] Low S, Chin MC, Ma S, Heng D, Deurenberg-Yap M (2009). Rationale for redefining obesity in Asians. Ann Acad Med Singapore.

